# Using machine learning prediction models for quality control: a case study from the automotive industry

**DOI:** 10.1007/s10287-023-00448-0

**Published:** 2023-03-16

**Authors:** Mohamed Kais Msakni, Anders Risan, Peter Schütz

**Affiliations:** Department of Industrial Economics and Technology Management, Norwegian University of Science and Technology, Torgarden, 7491 Trondheim, Norway

**Keywords:** Neural network, Random forest, Quality control, Manufacturing, 68T07, 68T09, 62H10

## Abstract

This paper studies a prediction problem using time series data and machine learning algorithms. The case study is related to the quality control of bumper beams in the automotive industry. These parts are milled during the production process, and the locations of the milled holes are subject to strict tolerance limits. Machine learning models are used to predict the location of milled holes in the next beam. By doing so, tolerance violations are detected at an early stage, and the production flow can be improved. A standard neural network, a long short term memory network (LSTM), and random forest algorithms are implemented and trained with historical data, including a time series of previous product measurements. Experiments indicate that all models have similar predictive capabilities with a slight dominance for the LSTM and random forest. The results show that some holes can be predicted with good quality, and the predictions can be used to improve the quality control process. However, other holes show poor results and support the claim that real data problems are challenged by inappropriate information or a lack of relevant information.

## Introduction

The emergence of the fourth industrial revolution, Industry 4.0, is primarily driven by advancements in information, communication, and intelligence technologies that can improve production flexibility, efficiency, and productivity in industry (Ibarra et al. 2018). While the definition of Industry 4.0 is broad, there are several key concepts associated with it, such as smart factories, the Internet of Things (IoT), cloud computing, cyber-physical systems, and Big Data Manufacturing (Santos et al. 2017). IoT technology enables to connect manufacturing resources, like sensors, machines, and other equipment, enabling interconnection between components and reducing human intervention. This also allows real-time, high-accuracy monitoring of product quality, equipment, and production processes. Real-time data flow can help identify problems early on and provide better visibility into the flow of materials and products. In addition, cloud computing makes data available to other systems with powerful resources, such as servers, storage, and software (Lee and Lee 2015). As many manufacturers have large amounts of data that go unused, cloud computing is seen as a way to transform the traditional manufacturing business model into an effective collaboration, helping manufacturers align business strategies and product innovation and create smart networks (Xu 2012). The amount of data collected from various systems and objects is growing at an exponential rate and is commonly referred to as Big Data. This concept is characterized by high dimensionality and high complexity due to the variety of formats, semantics, and quality of sensors and processes generating the data (Wuest et al. 2016). As a key concept in smart factories, Big Data can impact Industry 4.0 in three ways: enabling self-diagnosis, forecasting, and control (Tao et al. 2017). Conventional data processing software and technologies cannot fully leverage the potential of these large and complex datasets, and advanced methods such as machine learning algorithms are needed to organize and derive value from the data.

In the context of Industry 4.0, machine learning has been applied to different levels of the industrial process, such as anomaly detection, process optimization, predictive maintenance, quality control, diagnosis, and resource management (Roblek et al. 2016). Machine learning is seen as a promising improvement in manufacturing as it allows for decentralized, autonomous, and real-time decision-making without human interaction. It has the advantages of addressing large and complex processes and enabling continuous quality improvement (Dogan and Birant 2021). Unlike conventional algorithms, machine learning algorithms can dynamically learn from the system and automatically adapt to changes in the environment. It can also detect patterns and implicit knowledge from the data, improving existing processes and methods in manufacturing (Wuest et al. 2016). However, the application of machine learning is not straightforward. The performance of these algorithms can be hindered by the acquisition of relevant data in terms of volume and quality. On the one hand, the training data must be sufficiently numerous to reach the level of generalization, for which the learning model also performs well on new (unseen) data. On the other hand, the data may either contain inappropriate and redundant information or lack relevant information, as not all data is captured during the manufacturing process, and some attributes may not be available. Data preprocessing, which includes selecting relevant inputs and normalizing the data (Wuest et al. 2016), is also an important step before learning. The challenges of machine learning are not only limited to data but also include the algorithm itself. Some machine learning algorithms are more appropriate for specific applications, and the performance of some of them depends on selecting suitable hyperparameter settings. Despite these challenges, machine learning algorithms have the capacity to extract new information and provide better results than conventional algorithms.

One of the advances offered by Industry 4.0 is the opportunity to improve quality control in manufacturing. Traditionally, manufacturers have used Statistical Process Control (SPC) to ensure that product features are defect-free and meet specifications. SPC is based on the statistical assumption that random factors, such as humidity, temperature changes, and variations in raw material, tend to form a normal distribution centered on the quality characteristics of the product (e.g., length, weight, and hardness). Thus, the process is under statistical control, which allows for analyzing the outputs and the capability of the process. SPC provides tools and techniques for monitoring and exploring the process behavior and identifying anomalies (Tao et al. 2017; Oakland and Oakland 2018). With the technological capabilities of Industry 4.0, SPC can be supplemented to improve quality control further. Big data and cloud computing can use real-time data to detect quality defects and process instability at an early stage. For example, Gokalp et al. (2017) describe real-time data analysis to self-calibrate a process when a deviation in the trajectory of an ongoing machining process. In addition, machine learning can use time-series data of process and product variables to identify patterns and detect early process deviations so that preventive measures can be taken and the production process is stabilized.

This paper investigates the use of machine learning algorithms to predict product quality in manufacturing in order to support quality control. The focus is on bumper beams, which are an essential component of automotive crash management systems and are subject to strict quality control. The goal is to improve the quality control process in production by predicting the quality of future products, allowing for early adjustments, and reducing scrap production and downtime in the production system. The machine learning algorithms used in this study are based on neural networks and random forests. They are trained on historical data consisting of previously produced and measured parts provided by the manufacturer. The effectiveness of the neural network and random forest models is compared and evaluated for their ability to predict key product characteristics important for quality control. This work differs from previous research in that it develops machine learning models that use previously measured products to predict the quality of the next product rather than using the real-time state of the system to predict the quality of the current part.

The outline of the remainder of this paper is as follows. Section 2 discusses machine learning for quality control in manufacturing systems and presents related works in the literature. Section 3 introduces the concept of time series and relates it to process control and machine learning prediction models. The case study of this paper is discussed in Sect. 4. Section 5 shows the implementation and the obtained performance of the learning models. Finally, Sect. 6 gives a conclusion to this paper.

## Related works

Maintaining high-quality products and processes is essential for success in a competitive environment. In manufacturing, product quality is related to the functional aspects of the product that must be out of defects and out-of-tolerance conditions. The process of ensuring that any manufactured product meets the requirements is called quality control. If a product does not satisfy the requirements, it is considered a poor-quality product and will be removed from the production line. Many factors can cause quality to vary in the production process, such as humidity, temperature, and variations in raw materials and tools.

As technology advances and data becomes more available, new ways to perform more accurate, real-time quality control are emerging. Machine learning has already been successfully applied to tasks involving quality control and quality assessments and is expected to further improve the field of quality control in the future (Wuest et al. 2016). Collected data can be analyzed by learning algorithms in two ways (Tao et al. 2018). The first is to monitor the process in real-time to ensure product quality; for example, a deviation in tool trajectory can be detected using real-time analysis, and the process can be adjusted according to the requirements. The second is to identify emerging problems. Using historical data, the learning algorithms can identify patterns or predict the output characteristics of a process, enabling early detection of faulty products.

The neural network is one of the most widely used machine learning algorithms for process and quality control in manufacturing environments. Most of the applications are related to real-time analysis for process control or detection of defective products by image recognition. Karayel (2009) uses a feedforward neural network as an observer for a control system by predicting surface roughness in a computer numerical control (CNC) lathe. The prediction model uses process parameters as input data, i.e., cutting depth, cutting speed, and feed rate, to predict surface roughness. This prediction is later sent to the controller to determine the best cutting parameters for the CNC turning system. A similar network structure is used by Tsai et al. (1999) to predict real-time surface roughness in milling cutting operations. The model uses process parameters that consist of vibration measures, depth of cut, speed of the axis, and rotation and feed speed. Martin et al. (2007) propose a supervised feedforward neural network to replace a human expert in the quality control process of resistance spot welding. The machine learning model uses ultrasonic oscillograms to classify the quality spot welds into one of the six predefined levels. Zhao et al. (2020) use power signals to predict the nugget diameter from spot-welded joints in a real-time prediction system. The paper compares the performance of a regression model and feedforward network in monitoring weld quality and shows that the latter model provides better performance. To detect defects in parts, Wang et al. (2018) propose a deep convolutional neural network that uses raw images of flat surfaces to automatically extract product features for defect detection and improve production efficiency. For geometrically complex products (such as turbo blades), Wang et al. (2020) develop a similar model architecture in a cloud-based platform to meet the high-speed performance required by complex product images. Risan et al. (2021) develop a feedforward neural network to predict the location of a milled hole in a bumper beam.

Other works in the literature focus on prediction models to support quality control using random forest algorithms. With respect to the machining process, most works use process parameters as input variables for prediction models such as feed rates, tool wear, and drive power. Bustillo et al. (2021) investigate the prediction of surface flatness deviations in a face milling process using different machine learning algorithms. The input data consists of tool life and wear and drive power. The problem is first designed and evaluated as a regression problem and then as a classification problem using discretized flatness levels. For the regression problem, the random forest is outperformed by the two artificial neural networks, a multilayer perceptrons network and a radial basis functions network. However, when the classification problem is considered, the random forest gives the most accurate predictions. Wu et al. (2018) develop a random forest model to predict the surface roughness in fused deposition modeling. The input data is mainly based on the temperature and vibration of the table and extruder. The model can predict the surface roughness of a printed part with very high accuracy. Bustillo et al. (2018) propose different machine learning algorithms for surface roughness and loss-of-mass predictions in machining processes. The models studied include regression trees, multilayer perceptrons, radio basis networks, and random forest. The experiments show that multilayer perceptrons achieve the best surface roughness prediction. However, the random forest has the advantage of being more suitable for industrial use in the absence of experts in machine learning as this model has a non-parametric property. Agrawal et al. (2015) develop a multiple regression model and a random forest model for the prediction of surface roughness during hard turning of a hardened steel piece. Both models use the same cutting parameters as input variables. The results show better surface predictions for the random forest model. Li et al. (2019) use an ensemble of machine learning models to predict the surface roughness for an extrusion-based additive manufacturing process. A random forest is used to reduce the input variable size to improve the computational efficiency and avoid overfitting. Then, an ensemble of different machine learning models is used for surface roughness prediction.

Quality control can also be enhanced using time series data and prediction models. Ma et al. (2022) proposed a soft sensor model for quality prediction of industrial products using time series data and process features. The model framework is based on a neighborhood dimension reduction and a bidirectional gated recurrent unit. Shohan et al. (2022) use time series modeling to help improve the prediction quality of biofabrication process. Standard autoregressive time series models and machine learning models were tested. Experiments showed that the Long-Short Term Memory model provides the best performance in terms of mean square errors. In another application, Freeman et al. (2018) implement deep learning techniques in the prediction of air quality. The data consists of time series events of hourly air quality and meteorological events. Kim et al. (2022) use a descriptive time series analysis to predict downtime of a linear medical accelerator by using long-term maintenance data. Meng et al. (2022) deploy a deep learning model using time series data of historical images and image recognition of plants. The objective is to improve the quality by helping have healthy plants with high yields. Prediction using time series data is not limited to quality improvement but covers a wide range of applications. Many works have recently emerged to predict COVID-19 transmission using time series and deep learning models (Long Short Term Memory networks and Gated Recurrent Units), e.g., Rauf et al. (2021); Ayoobi et al. (2021).

## Methodology

Time series data, which consists of observations recorded at specific times, is often available in manufacturing processes and equipment. It is then important to exploit these data to extract valuable information for the manufacturers. This task corresponds to finding a model that describes a time series. This model estimates the relationship between the variable of interest *Y* and the input variables *X* using a function *f*. While various approaches can be applied, i.e., physical, statistical, and machine learning models, the nonlinear and high-dimensional aspects of manufacturing systems make it very difficult to develop a satisfactory model for estimating *f*. Despite this challenge, developing a time series model has several advantages, such as a compact description of the time series, hypothesis testing, separation and filtering of noise from data, and time series prediction (Brockwell and Davis 2016).

### Times series and statistical process control

In the context of quality control, SPC is a widely used method that involves process capability and statistical analysis of process results. These methods rely on monitoring and analyzing the product features relevant to product quality. By using samples of a specific size from the process, causes for variation can be identified, and adjustments can be made (Groover 2019).

One of the primary techniques is the control chart, which offers a visualization way to study the evolution of a process over time. A time-series data is represented in a chart with a central line for the average, an upper line for the upper control limit, and a lower line for the lower control limit. These control limits are then compared to the actual data to see if the process variation is under control. When the process is under statistical control, the control limits are defined based on the process capability (*PC*), which provides information about the accuracy of a process’s performance over time and measures the ability of a process to meet its specifications (Oakland and Oakland 2018). It can be defined as:

1 $$\begin{aligned} PC=\mu \pm 3\sigma \end{aligned}$$

where $\mu$ is the mean of the process, and $\sigma$ is the standard deviation. Thus, 99.73% of outputs of a controlled process are within $3\sigma$ limits.

### Machine learning prediction models

The main challenge of machine learning models is to establish a valid representation of the input data (the input variables *X* of the time series) by performing some transformation (the model or function *f*) that can approximate the expected outcomes (the variable of interest *Y*). The provided data set is commonly referred to as the “training set" and is used by machine learning algorithms to apply a predefined set of operations to build a model. Thus, machine learning models are not explicitly programmed to make decisions or predictions but are created during the learning stage. Machine learning has been successfully applied to a variety of problems across different domains, such as image recognition, anomaly detection, and quality control. In this work, two classes of machine learning algorithms are developed, namely neural networks and random forests, to predict the location of holes in future products. Since the locations are continuous values and the training set is composed of input and output variables, the problem is referred to as a regression problem with supervised learning. A general description of neural networks and random forests is given in the following subsections.

#### Neural networks

**Fig. 1:**
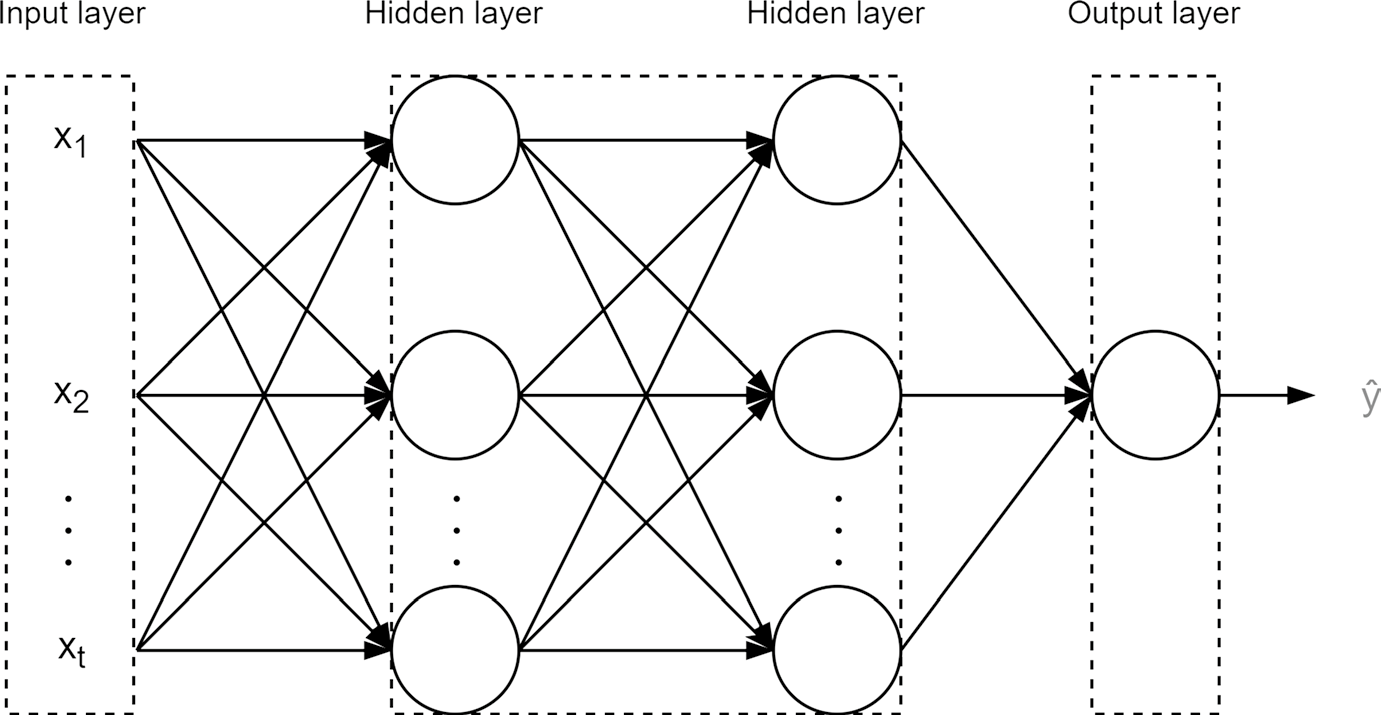
An example of a feedforward neural network with an input layer, two hidden layers, and one output layer with one target variable. Adapted from (Ketkar and Moolayil 2021)

Neural networks are one of the most commonly known machine learning algorithms, and they have been successfully applied to a wide range of fields. This learning algorithm is inspired by a biological network of neurons, in which neurons are chemically connected to form an extensive network. In artificial neural networks, neurons are modeled as nodes and connections as weights. The role of weights is to computationally activate or deactivate a connection between two nodes: A positive weight indicates an active connection, whereas a negative weight prohibits the link between the nodes. A node receives many connections (weights) that are transformed into a single output. Typically, the neurons in a neural network are organized in layers. The first (input) layer passes the input data to the network without any transformation, and the last (output) layer consists of output variables. The hidden layers connect the input layer to the output layer and perform the data transformation using activation functions. The role of an activation function in the hidden layers is to transform the weighted sum of the input into an output that will be used in the following layers. A general structure of the feedforward network is illustrated in Fig. 1.

The layered representation of neurons can represent complex relationships between input and output data and extract complex patterns. Indeed, neural networks can model non-linear-statistical data and handle high-dimensional and multivariate data. However, they require more data than other machine learning models, and the best performance requires extensive customization as neural networks depend on several hyper-parameters. Also, neural networks do not provide any information about how the outputs are computed, a problem commonly referred to as the black box in machine learning (Ian et al. 2016).

Although neural networks are well suited for a large variety of problems, such as image recognition and text recognition, they suffer from a major issue known as the vanishing gradient problem, which prevents learning long-term dependencies (Rehmer and Kroll 2020). This makes it difficult to train standard neural networks on long data series (Kinyua and Jouandeau 2021). The vanishing gradient problem can be addressed by including gated units, such as the Long Short-Term Memory and the Gated Recurrent Unit (Rehmer and Kroll 2020).

#### Random forests

**Fig. 2:**
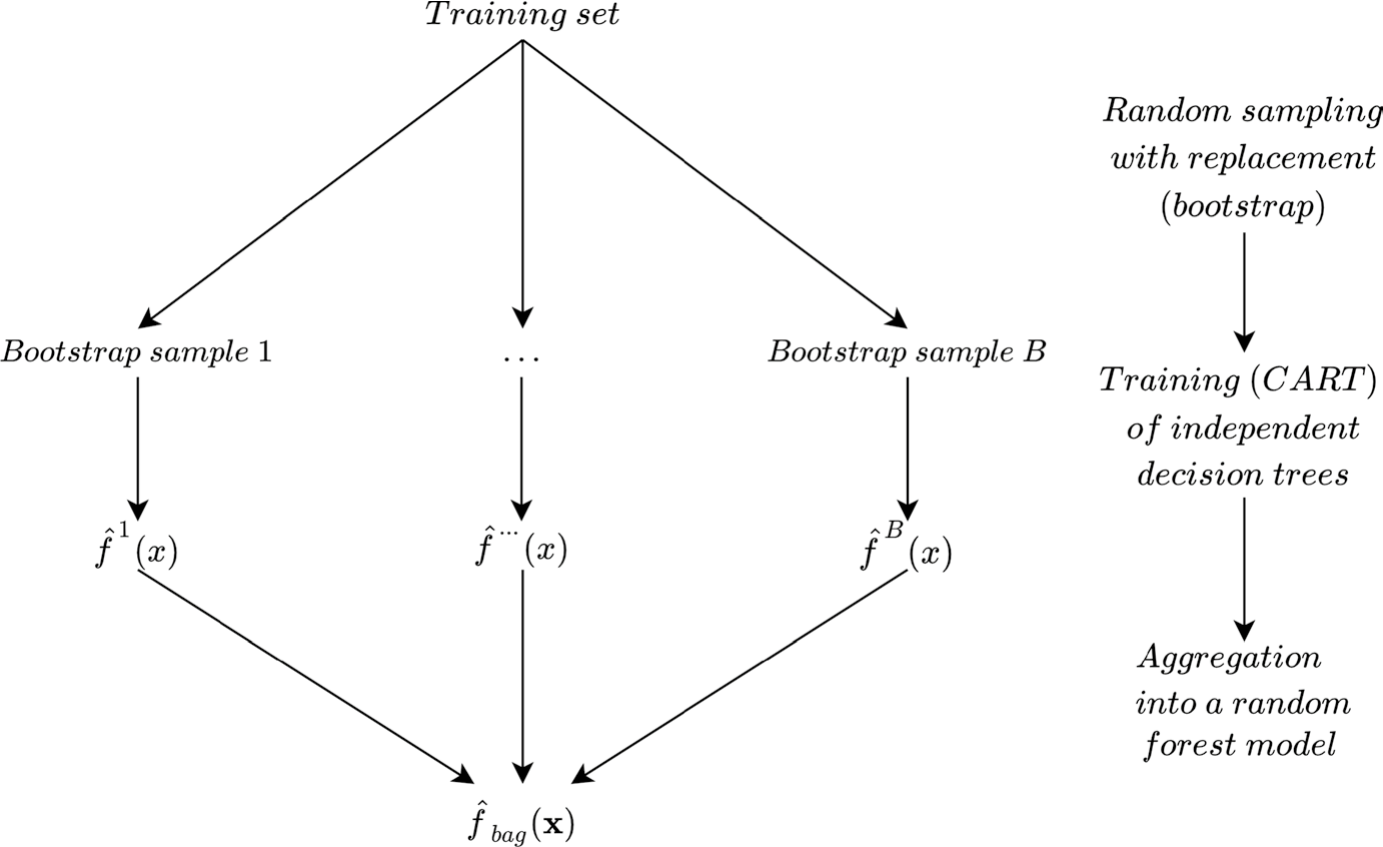
Flowchart of training a random forest tree and aggregation of results. Adapted from (Genuer and Poggi 2020)

The random forest algorithm has become a widely used machine learning algorithm because of its simplicity and accuracy (Biau and Scornet 2016) and its ability to perform both supervised and unsupervised learning, as well as classification and regression problems (Genuer and Poggi 2020). This algorithm is a statistical learning method proposed by (Breiman 2001), based on the principles of ensemble learning. In machine learning, ensemble learning refers to the techniques of combining the predictions of a group of trained models (an ensemble). The idea is that by aggregating the outcomes of several models, the prediction of the ensemble is more likely to perform better than any individual model in the ensemble. For the random forest, the algorithm is trained on different and independent training subsets (bootstraps) to obtain several models, referred to as trees. Figure 2 illustrates a general structure of the random forest.

A decision tree is a predictive model with a tree-like structure where the decision progresses from the root node through internal nodes until it reaches a leaf. A node corresponds to a binary split of the predictor space to continue the decision flow in one of the two sub-trees of the node. A leaf in the decision tree represents a predicted value or class label, and the path to the leaf represents the classification rules. Such a decision representation makes decision trees readable and simple to interpret. Although there are different algorithms for building a decision tree, the classification and Regression Tree (CART) algorithm is widely used for random forests (James et al. 2013).

For a classification problem, the decision tree uses the input values to reach one of its leaves and, here, to find the predicted class. Similarly, a regression problem uses the same decision tree structure, with the difference that the leaves correspond to continuous target values. To make the decision trees independent of each other, they are trained on B randomly drawn and independent subsets of equal size. Each subset is used to train B decision trees that will finally be aggregated into a forest.

One advantage of the random forest algorithm is that it does not require heavy computation for training. It is easy to tune as it depends only on a few hyper-parameters. Another advantage is that it is suitable for high-dimensional problems with multivariate data where the number of variables far exceeds the number of observations, and vice versa (Géron 2019). However, the prediction quality of the random forest is highly dependent on the quality of the training set, e.g., it cannot predict values outside the minimum and maximum of the values in the training set.

## A case study

The product studied in this paper is the bumper beam, a component of a crash management system in cars. The beam is formed from an extruded aluminum profile and is machined and cut before being assembled to the bumper with screws.

The beam is placed using clamps at predefined locations during the machining step. Then, the CNC machining starts with the milling of reference holes, which are of particular interest because they are used to locate and mill other holes by CNC machining. In total, there are 20 milled holes, each with narrow tolerance ranges regarding their location in the beam. Any displacement of the reference holes results in deviation of the connected holes. Quality control of the milled holes is performed after the machining process, when a new product is released, or at predefined intervals. The interval length between two quality controls is typically two hours and is considered by the manufacturer as satisfactory to guarantee high-quality standards while ensuring smooth production. During quality control, the geometric characteristics of all milled holes and the beam curvature are automatically measured in an XYZ grid system, resulting in a total of 144 different features. When the measurement report shows any deviation, the entire batch of products is rejected, and the production line is disrupted. The production batch since the last control is scrapped. An experienced operator makes the necessary changes to the machine settings. Then a new beam is machined, and another quality control is performed. The goal of the manufacturer is to reduce the downtime of the production as much as possible to minimize direct economic loss.

Many factors can cause variations in the CNC machining process, including both random variations such as clamping force, temperature, and variations in upstream activity, as well as assignable variations such as replacement of CNC parts and change in the beam type being processed. Unfortunately, not all of these variations are available to be considered as part of the input to the learning models.

**Fig. 3:**
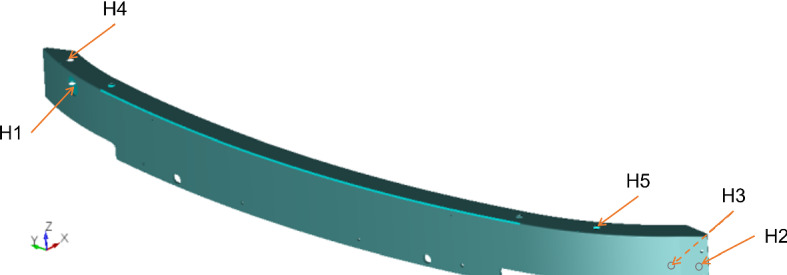
Illustration of the bumper beam shape and the locations of the five reference holes

Figure 3 illustrates the shape of the bumper beam and the locations of the reference holes. Two reference holes (H1 and H4) are located on the left side of the beam, and three other holes (H2, H3, and H5) are located on the right side of the beam. H1, H2, and H3 are located using the YZ coordinate system, whereas H4 and H5 are located using the XZ coordinate system. This work aims to improve the quality control of the milled holes by predicting the reference hole locations of the next product to be manufactured. Machine learning models are implemented to predict future hole positions, which can be used as a preventive measure to avoid out-of-tolerance products. Since this information is available, early adjustments can be made, and the production flow is smooth. The proposed learning models do not depend on real-time data, as is the case in many literature works, but consider a time series analysis that uses previous measures to predict the hole locations in the upcoming product. Historical data from all available measurements is used as input to train the models. The target variables are the coordinates of all reference holes.

**Fig. 4:**
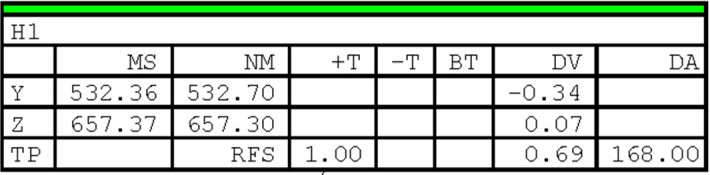
A table from a control report of the studied beam showing the measurements of the reference hole H1

Figure 4 shows an example of the measurements and data collected for the reference hole H1. This hole is located using a measured value (MS) and a nominal value (NM). The deviation (DV) of H1 is the difference between MS and NM. Based on the deviations of *Y* and *Z*, denoted here by *dy* and *dz*, the true position (TP) of the measured hole can be computed. The TP defines a circular tolerance area for the hole position and is defined by Eq. 2.

2 $$\begin{aligned} TP=2\sqrt{dy^2+dz^2} \end{aligned}$$

**Fig. 5:**
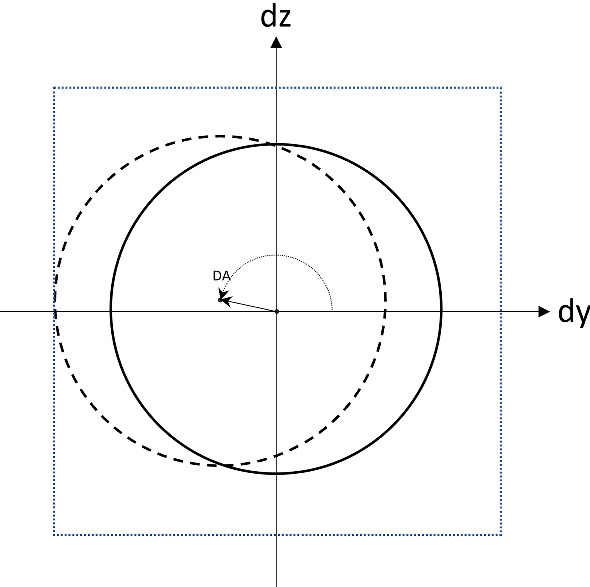
An illustration of the displacement of H1 of Fig. 4. The related milled hole is represented by a dotted circle. The solid circle corresponds to the nominal position, and the dotted square represents the area where the hole meets the specifications. *DA* shows the angular deviation of the milled hole

For the example of Fig. 4, the TP measure is within the predefined tolerances limits ($-T$ and $+T$). The angular deviation (*DA*) complements the TP measure and provides information about what direction the hole has moved. The actual location of H1 related to this example is represented by a dotted circle in Fig. 5.

The TP values of the other holes are calculated similarly using the deviations of the two coordinates locating a hole. The decision as to whether a hole location is within specification or not depends on the TP values, which must be within the lower and upper limits defined by the manufacturer.

## Experiments and results

In this section, the data, implementation, and performance of each machine learning model are discussed.

### Training, validation and test set

**Fig. 6:**
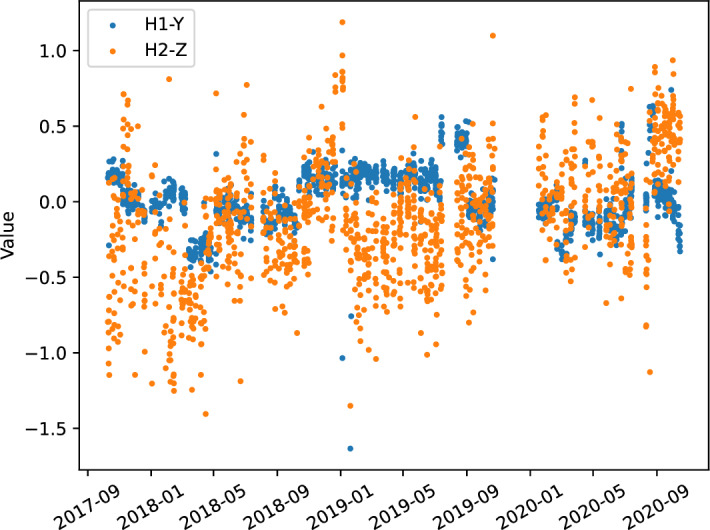
Scatter plot of the measurement values of H1-Y and H2-Z over the data collection period

The dataset used for the quality control prediction consists of 1255 measurement reports, covering three years. Each report includes a timestamp of the measurement operation, the locations of the 20 milled holes, and the curvature of the beam, resulting in 144 different point measurements. It should be mentioned that the interval between two quality control measurements is not always two hours and can vary greatly depending on the production schedule, holidays, priorities, etc. As shown in Fig. 6, which depicts the measurements of two hole-coordinate pairs using a time-stamped axis, the production for the bumper beam under study was partially interrupted during November and December 2019. This kind of interruption can be found several times (about 15 times) throughout the dataset, with most of them lasting for one or two weeks. Despite these interruptions, we assume that the dataset is continuous as the number of interruptions is small, and the machine learning models used in this study depend only on lag features for prediction.

For a given learning algorithm, the variable to be predicted (the output) is a single hole-coordinate pair, e.g., H1-Y, that is trained and tested separately. The prediction of measure *t* uses all points of the three lagged measurements, i.e., $t-3$, $t-2$, and $t-1$, as input, resulting in $3\times 144$ independent variables for every variable to predict. Indeed, preliminary testing showed that the prediction mainly depends on the last observation $t-1$, and that it can be slightly improved by integrating three-lagged input. Furthermore, it should be stated that all measures are related to relative deviation. These values are available with the raw data used in this study.

The dataset is divided into two subsets. The first 70% of the dataset is used to train and validate the machine learning algorithms, and the remaining 30% is used to test the prediction performance of the models. Since the default hyperparameters of all models cannot guarantee optimal results for the prediction problem, we performed a hyperparameter tuning step that was done on the first subset of data. This subset was, in turn, divided into two other subsets, where the 50% of the dataset is used for training, and the next 20% is used for validation. The hyperparameters test was done on a randomly chosen hole, namely H5-X (the results of subsection 5.4 show that this hole has an average performance). The best parameters found were then used for the other holes. It should be noted that other holes were selected for the hyperparameter tests, and similar results were obtained.

### Implementation

In addition to the random forest, two neural network models are considered for prediction purposes. The first model is based on a standard neural network, hereafter referred to simply as a ‘neural network’, and the second is a Long-Short Term Memory (LSTM). All machine learning models were implemented in Python 3.8.6 using the Scikit-Learn library (for the neural network and the random forest) and the Keras library (for the LSTM). The input data is processed using Pandas 1.1.3 and Numpy 1.19.2, and the visualization tools are based on Matplotlib 3.3.2. The working environment is Jupyter Notebook on a Windows machine with a Core i7 CPU and 32 GB of RAM.

The parameters of the neural network are one hidden layer of 100 neurons, and the LBFGS solver is used as the identity activation function. The hyperparameter function of the random forest is used to find the best parameters, with the best results obtained with the default settings. A greedy hyperparameter tuning was performed for LSTM, where the number of epochs ranged between 1 and 1000, the batch size was set to 1, 2, and 4, and the number of neurons was set to 1, 2, 4, and 10. The best parameters were found for 100 as the number of epochs, two as the number of batches, and one as the number of neurons.

Finally, it should be mentioned that the neural network and LSTM models are more sensitive to data scaling than the random forest. The input data is pre-processed by standardizing, and the same scaling is then applied to the input of the test set.

### Data analysis

The first step in analyzing the collected data is to understand the problem and verify its quality. This step involves visualizing and evaluating the relevance of the data, identifying outliers, and removing bad entries. Figure 6 presents the different measured values for the hole-coordinate pairs H1-Y and H2-Z over the data collection period. It can be observed that the H2-Z measurements are more spread out than H1-Y measurements. In contrast, H1-Y measurements fall within a narrow range that varies over time without a distinctive trend (e.g., degradation over time). These variations could potentially be explained by changes in the production process, but without additional data, it cannot be confirmed. Furthermore, the dispersion of the values in Fig. 6 (especially for H1-Y) supports the idea of using lagged measurements for prediction purposes.

**Fig. 7:**
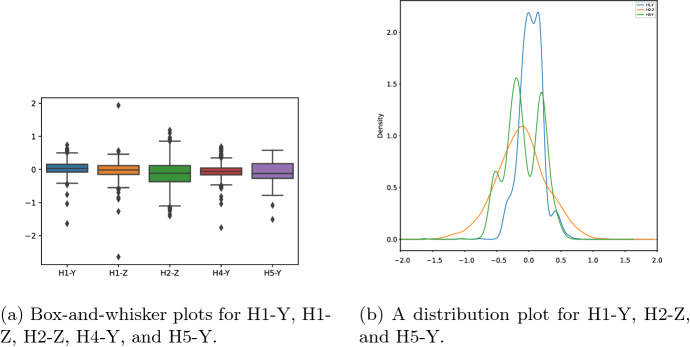
Analysis of measurement data for a subset of reference holes and coordinates

Figure 7a illustrates the distribution of the different measurements of hole-coordinate pairs. Since showing all pairs in the same figure makes it difficult to read, we restrict the representation to only five variables that are selected randomly and have data properties compared to other variables. Figure 7a indicates that all variables share similar data distribution properties. The median is almost equal to zero, and the interquartile ranges (IQRs) presented by boxes are very narrow, revealing that the measurements are concentrated within this area, especially for H1-Y, H1-Z, and H4-Y. In addition, Fig. 7a shows for each pair the outliers, defined as the points outside of the whiskers, at 1.5 of IQR from the first and third quartiles. We can see that the majority of outliers are condensed at the head and tail of whiskers, and very few measurements are far from the rest of the data. Thus, the data available for this study is considered to be of good quality and does not require preprocessing.

Furthermore, Fig. 7b illustrates the distribution plots of pairs of hole-coordinate. For the sake of readability, we select only three representative pairs as they have similar distributions to the other holes. The H2-Z plot shows a normal distribution that is symmetric and bell-shaped but slightly deviated from the zero mean. That is, this particular pair of hole-coordinate can be represented by a standard normal distribution. As for the H1-Y measurements, the distribution has a slight multimodal shape that can be smoothed to a normal distribution with high kurtosis, meaning that most observations have zero deviation. However, H5-Y has different peaks with different densities, which means that most measurements are slightly deviated from zero.

### Performance of the models

In this subsection, the performance of the neural network, LSTM and random forest are assessed from both a quantitative and qualitative perspective. In addition, the models are compared to a standard autoregressive time series model.

#### A quantitative comparison

Three common performance metrics for regression problems are used to evaluate the predictive quality of models. The first is the Mean Absolute Error (MAE) which shows the magnitude of the overall error between the observed and predicted values. It neither eliminates the effect of positive and negative errors nor penalizes extreme forecast errors. The second is the Mean Squared Error (MSE), which penalizes extreme values. A high value of MSE shows a significant deviation between observed and predicted values, whereas a low value indicates that the predicted values are very close to the observations. Finally, the root mean square error (RMSE) is commonly used for regression problems and measures the square root of the second sample moment of residuals. RMSE is used to compare prediction errors of different models for the same data set and a particular variable, as it is scale-dependent. The definition of these three metrics are given in Eqs. (3), (4), and (5).

3 $$\begin{aligned} \text {MAE}=\frac{1}{n}\sum _{t=1}^{n}\Vert y_i-{\hat{y}}_i\Vert \end{aligned}$$

4 $$\begin{aligned} \text {MSE}=\frac{1}{n}\sum _{t=1}^{n}(y_i-{\hat{y}}_i)^2 \end{aligned}$$

5 $$\begin{aligned} \text {RMSE}=\sqrt{\frac{1}{n}\sum _{t=1}^{n}(y_i-{\hat{y}}_i)^2} \end{aligned}$$

where,

- $y_i$ is the observed target value,
- ${\hat{y}}_i$ represents the predicted target value, and
- *n* is the number of observations.

**Fig. 8:**
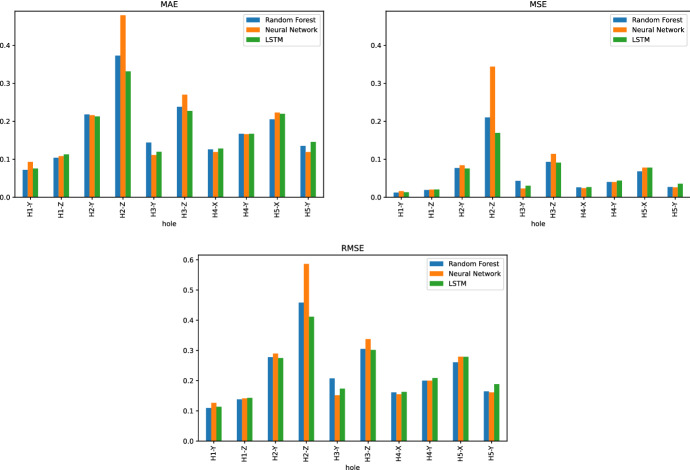
Comparison between the Random Forest, Neural Network and LSTM models using the MAE, MSE and RMSE metrics for the locations of all reference holes

Figure 8 shows the MAE, MSE, and RMSE metrics for the Random Forest and the two versions of Neural Network. Except for the reference hole H2, all models can provide reasonable predictions relative to the actual observations, i.e., the MAE metric ranges from 0.11 to 0.28 mm for the other holes. In particular, the predictions for hole H1 provide the best metrics, and H2 has the worst prediction metrics for the Y and Z coordinates. When learning models are considered, LSTM provides the best performance for H2-Z, where the metric MAE is improved by 31% over the neural network. For the remaining hole-coordinate pairs, the average MAE is the same for all models, i.e., 0.16. However, Random Forest and LSTM perform slightly better than Neural Network for MSE, i.e., 0.045 against 0.047.

**Fig. 9:**
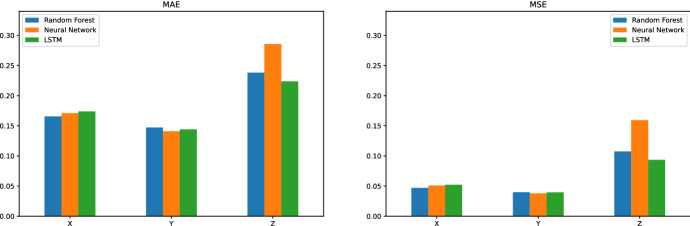
Comparison between the prediction models by grouping the holes located in the same coordinate (X, Y or Z). The metrics are MAE and MSE

Figure 9 groups the hole-coordinate pairs that are located in the same direction and provides the MAE and MSE metrics of each direction by learning model. Together with the results shown in Fig. 8, we observe that for holes in the YZ coordinates (H1, H2, and H3), all prediction errors in the Z coordinate are higher than the corresponding Y coordinate. The same pattern appears for holes in the XY coordinates (H4 and H5) where the prediction errors in the X direction are slightly higher than in the Y direction. Since the comparison by coordinate is consistent for all learning models, it can be concluded that the models are better suited to one direction than another. Furthermore, when considering the locations of the holes in the beam, it can be observed that the holes on the left side of the beam, i.e., H1 and H4, are better predicted than the holes on the right side of the beam, i.e., H2, H3, and H5.

**Fig. 10:**
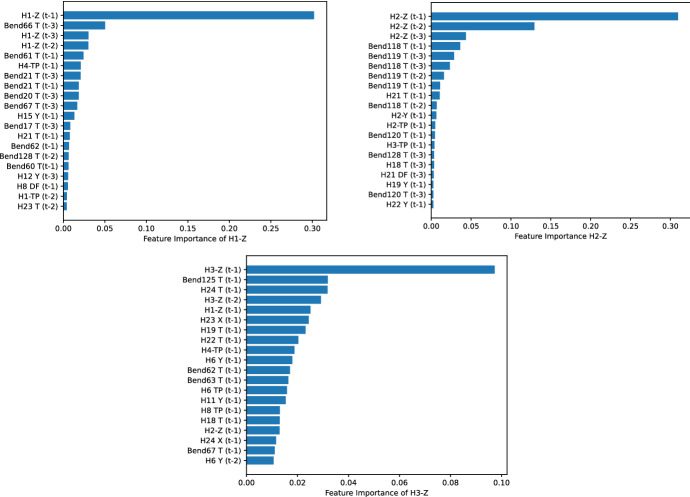
Top 20 most important features of the random forest model for the hole-coordinate pairs in the Y direction (H1-Z, H2-Z and H3-Z)

The previous analysis is further extended to include the feature importance of the predicted hole-coordinate pairs in the Y direction. The metrics of H2-Z and H3-Z show poor predictions compared to H1-Z, despite all of them being located in the same direction. Therefore, it is worth exploring which variables are the most significant and which factors have the greatest impact on the prediction. This can be done by analyzing the average feature importance of the decision trees in the random forest model. Figure 10 shows the top 20 most important features for H1-Z, H2-Z, and H3-Z. It can be observed that there is a variety of variables involved in the importance feature, including the previous measurements of the hole to be predicted, with the lagged time in parenthesis, as well as bend and other (non-reference) hole measurements. In the label in front of a variable name, the letter *T* refers to the twist tolerance for a bend measurement. The same letter is also used for small holes and indicates the distance to a reference hole (a specific metric set by the manufacturer is used). Lastly, the *DF* denotes the diameter of a milled hole.

Figure 10 shows that three factors affect the prediction quality. First, all predicted pairs depend strongly on their immediate previous measurement $t-1$ and, to a lesser extent, on $t-2$ and $t-3$. However, basing the predictions solely on previous measurements leads to poor prediction metrics, as can be seen in H2-Z. Second, a diverse range of information leads to better predictions. The feature importance of H1-Z shows that different sources of information are used, i.e., Bend 66 and Bend 67 are bend measurements near the location of H1, Bend 61 is in the middle of the beam, and Bend 20 and Bend 21 are on the other side of the beam. Third, having only low-importance values does not help to have good predictions (i.e., H3-Z). This indicates that the learning model cannot identify the relevant features for a good prediction of the target variable. Overall, this analysis confirms the importance of considering all available information and three-lagged measurements for prediction purposes.

#### A qualitative comparison

**Fig. 11:**
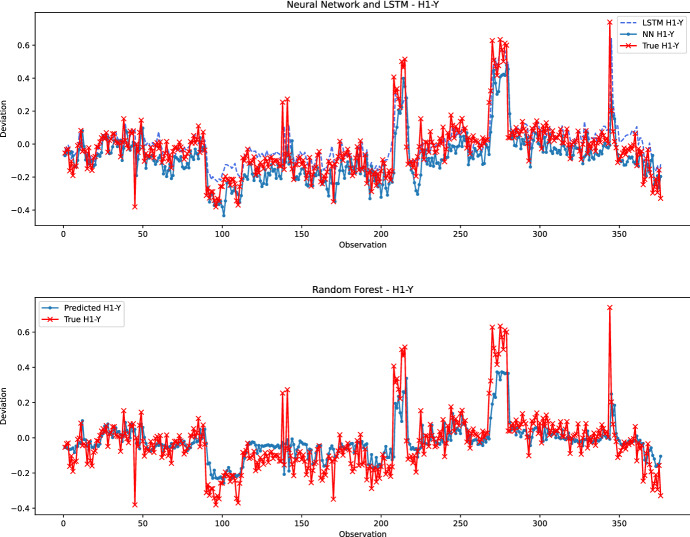
Prediction performance of the neural network (top) and random forest (bottom) models for the best-predicted coordinate – H1-Y

Figure 11 gives a qualitative comparison of the prediction models for the best-performing hole-coordinate pair, namely H1-Y. The bottom part of Fig. 11 plots the actual values and those predicted by the random forest, while the top part draws the predictions of Neural Network and LSTM (since they belong to the same family of learning models) together with the actual values. It can be observed that, in general, the random forest provides restricted and smooth predicted values. This observation is notable for the first and last segments of observations, where the values predicted by the random forest are always within the fluctuations of the actual measurements. However, LSTM and neural network models can track the spikes better to generate predictions as high as the actual values. Except for the measurements around Observation 100, LSTM performs marginally better than the neural network. Overall, all H1-Y predictions can be considered of high quality with good performance for both the random forest and LSTM.

**Fig. 12:**
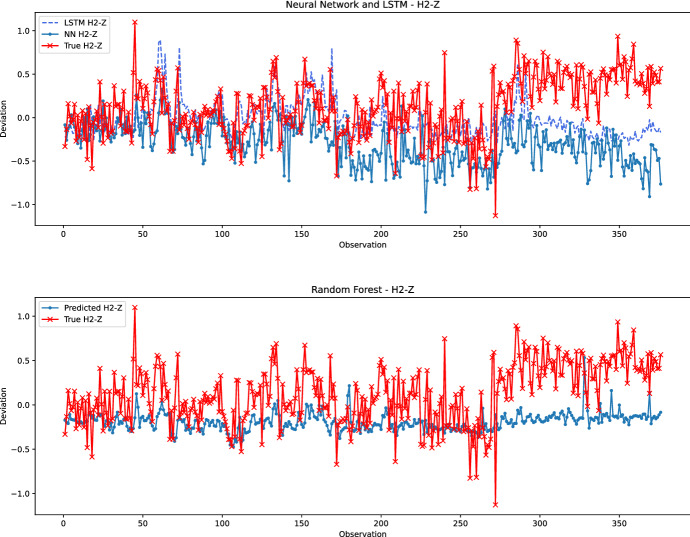
Prediction performance of the neural network (top) and random forest (bottom) models for the worst predicted coordinate – H2-Z

In the second qualitative comparison, Fig. 12 illustrates the performance of the prediction models for the worst-performing hole-coordinate pair, namely H2-Z. It can be seen that there is a significant gap between the actual observations and the predicted values for all models. Except for the first 40 observations, the random forest and neural network models generate poor predictions compared to the actual values. For the interval between observations 40 and 270, the neural network attempts to follow the trend of the actual values without providing good predictions, while the random forest predicts deviations close to zero. However, the LSTM shows much better performance as the actual values are better tracked. Indeed, when this segment of observations is considered, the MAE of LSTM is 0.24 against 0.36 and 0.30 for the neural network and random forest, respectively. This explains the better metrics obtained by LSTM for H2-Z. From about observation 270, the deviation of the predicted values from the actual observations becomes increasingly significant for all models. In particular, the random forest generates a prediction close to zero. We can conclude that this last segment is very peculiar; unknown changes have been made to the production process preventing the learning models from making good predictions. The limited performance is not due to limited learning capacity but rather to missing information not provided to the models. Indeed, as previously discussed and shown in Fig. 8 and Fig. 9, the prediction is better in one direction than in another. Similarly, the prediction of the holes located on the left side of the beam is better than the holes located on the other side of the beam. This may be due to the clamping forces applied to the beam during the machining process, which is not available for this study. Another reason may be a variation in the upstream activity, for example, when the aluminum profiles are bent.

#### Comparison with an autoregressive time series model

The performance of the machine learning models is compared to an autoregressive integrated moving average (ARIMA) model. The model was fitted to the time series data to predict future points in the series. We recall that ARIMA is a univariate model, which means that previous data of a specific hole-coordinate pair is used to predict the next observations. The ‘statsmodels’ library was used to implement the ARIMA model, and the pre-built ‘auto_arima’ function was called to identify the most optimal values for trend elements (*p*, *d*, *q*), where *p*, *d*, and *q* are the trends in autoregression order, difference order, and moving average order, respectively. The best parameters differ from one hole to another, for example, the ARIMA (3,1,3) model is used for H1-Y, and the ARIMA (2,0,4) is used for H2-Z.

**Fig. 13:**
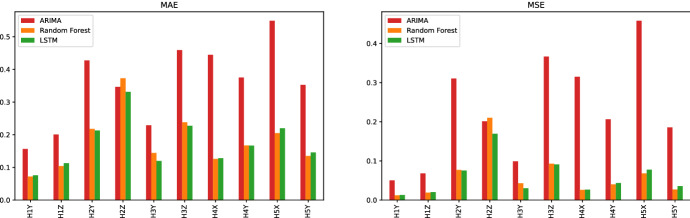
A comparison between ARIMA, random forest, and LSTM using MAE and MSE metrics

The metrics of the ARIMA models are shown in Fig. 13 and compared to the random forest and LSTM models. To compute the MAE and MSE, a static prediction for ARIMA for all pairs is used, meaning that the fitted models employ the actual value of the lagged dependent variable for prediction. It is clear that the learning models perform much better than the ARIMA models for all holes. This indicates that integrating other information such as bend and other holes’ measures help to achieve a better prediction. The exception is for H2-Z where ARIMA performs better than the random forest and is comparable to LSTM. This can be explained by the fact that ARIMA provides a very restrictive prediction that is always within the bounds of the actual values, which helps to reduce the error, especially for the last observation segment for H2-Z. However, as shown in Fig. 12, the random forest and LSTM perform poorly for this last segment, starting at observation 270. Despite this poor performance, the learning models provide important information for this particular segment. They indicate that unknown changes have been made to the production process. This information cannot be derived from the ARIMA results.

### Residual evaluation

**Fig. 14:**
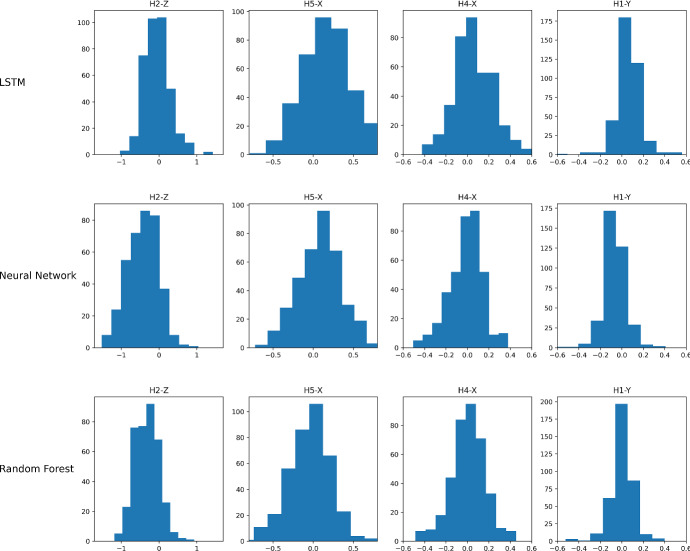
Residual normality test for the predictions of LSTM, Neural Network, and Random Forest for the worst and best MAE metric, H2-Z and H1-Y, respectively. The comparison also includes H5-X and H4-X ranked 7th and 3rd on the same MAE metric

The prediction quality of the proposed models can be further assessed by analyzing the residuals, which are the difference between the actual and predicted values. These residuals should be uncorrelated and normally distributed with zero mean (Kuhn and Johnson 2013). Figure 14 shows the histograms of residuals for the hole-coordinate pairs studied above, namely H2-Z and H1-Y, for all models. In addition to the worst- and best-predicted coordinates, the analysis includes H5-X and H4-X, which are ranked around 7th and 3rd positions in terms of MAE for all models considered.

The histograms in Fig. 14 provide insight into the distribution of residual errors and the prediction quality. In particular, the distribution of residuals for H2-Z (the worst-predicted coordinate) is non-Gaussian and positively skewed with high kurtosis (large tails) for the neural network and random forest. This confirms the poor prediction quality for these two prediction models. However, LSTM shows a better distribution of residuals for H2-Z, which confirms the better performance obtained for this coordinate compared to the other two prediction models. For the H1-Y coordinate, the distribution of residuals shows a mean close to zero and a low kurtosis. The histograms confirm the strong performance obtained with the H1-Y coordinate. As for H5-X and H4-X, the visualization of the residuals is close to the Gaussian distribution, especially for H5-X with the neural network. With the mean of the distribution for H5-X and H4-X being almost equal to zero for all models, it can be concluded that the predicted and actual values are not correlated. The prediction performance of the learning models is generally good for some hole-coordinate pairs.

### Confidence bounds and TP-outliers detection

**Fig. 15:**
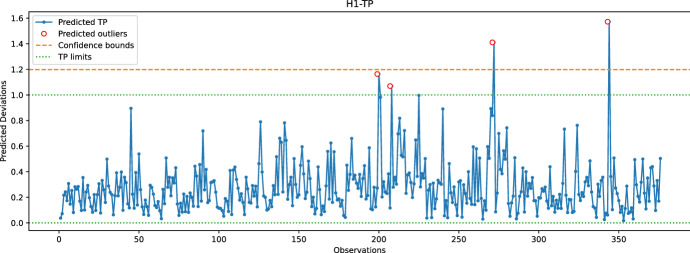
Predicted TP values for the hole H1 using the random forest model with a $3\sigma$ level. The TP limits for H1 are between 0.0 and 1.0 as set by the manufacturer

Figure 15 shows the predicted TP values for the hole H1 (discussed in Sect. 4), along with the corresponding $3\sigma$ level (shown with orange dashed line) and the TP limits set by the manufacturer (between 0.0 and 1.0). The predicted TP value is calculated according to Eq. 2 and is based on the coordinates H1-Y and H1-Z predicted by the random forest model. This figure aims to assess whether the predicted TP values are within statistical control and whether they can be used for quality control of the bumper beam. The validation test is used for the $3\sigma$ control. As can be seen, the TP limits are stricter than the $3\sigma$ level. Only two outliers out of four are above the confidence bound. When the remaining holes are considered, a similar observation is obtained, i.e., the TP limits and $3\sigma$ are almost the same. The only exception is for the hole H4, for which the $3\sigma$ level is stricter than the TP limit; however, this can be considered acceptable as only one observation of H4 deviates from both TP and $3\sigma$ limits. In conclusion, the process variation is under control and subject to random factors.

**Fig. 16:**
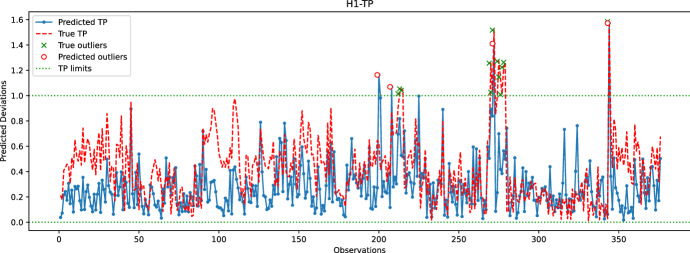
Comparison between the predicted and true TP values for the hole H1. The random forest model is used to predict the coordinates of H1

**Fig. 17:**
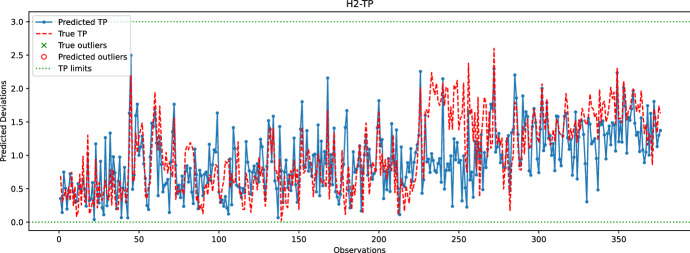
Comparison between the predicted and true TP values for the hole H2. The random forest model is used to predict the coordinates of H2

**Fig. 18:**
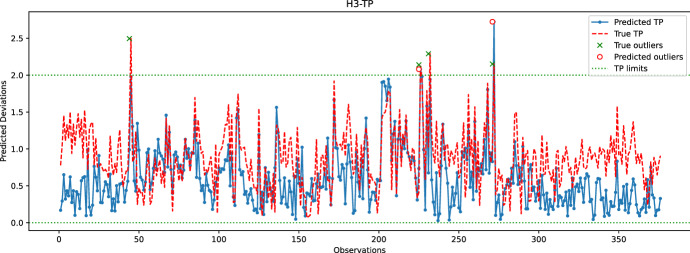
Comparison between the predicted and true TP values for the hole H3. The random forest model is used to predict the coordinates of H3

**Fig. 19:**
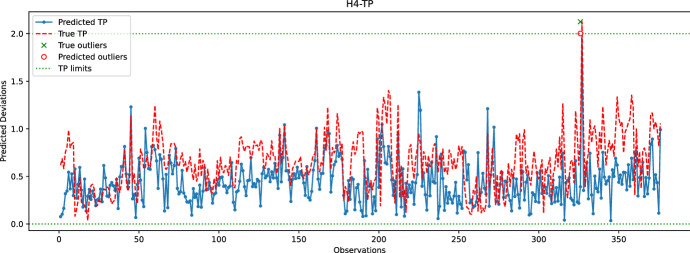
Comparison between the predicted and true TP values for the hole H4. The random forest model is used to predict the coordinates of H4

**Fig. 20:**
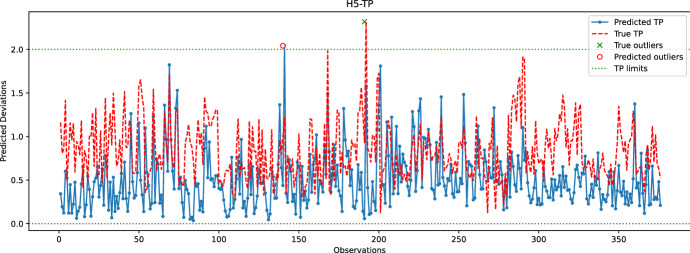
Comparison between the predicted and true TP values for the hole H5. The random forest model is used to predict the coordinates of H5

In the last experiment, Figs. 16, 17, 18, 19 and  20 compare the real and predicted TP values for holes H1–H5. The TP limits of each hole, as set by the manufacturer, are highlighted with a dashed green line. These figures show a difference between the actual and predicted TP values. The results of the prediction model cannot follow the fluctuations of the actual TP values. However, when it comes to outliers, the model can give some insights into when the deviations might occur. For the hole with the best-predicted coordinates (H1, shown in Fig. 16), the predicted TP values are located in the same observation area where actual TP deviations are observed. The first area, located around observations 210-213, is detected in advance by the prediction model before observation 210. The second area records consecutive actual TP measures that exceed the upper limit. Although the prediction model cannot detect all of these outliers, it has a prediction located in this area. The last area includes only observation 344, which is perfectly detected by the prediction model with a similar actual TP value.

The same statement is also valid for H3, with the deviations of observations 226 and 272 being correctly reported by the model. Although the deviation of observation 45 is not detected, the predicted value of TP is very close to the upper limit, which may indicate that an early adjustment of the CNC machining settings should be made. As for H4, only one deviation is reported that is perfectly predicted by the model. While the results are satisfactory for the holes discussed above, the prediction of TP outliers for H5 is poor. The predictions of the X and Y coordinates of H5 are among the worst, which may explain the poor quality of the prediction of TP values for this hole. Finally, no conclusions can be drawn from the analysis of the H2 results because there are no outliers for this set of observations. However, it is unlikely that the prediction model would be able to identify outliers for H2, as the H2-Y and H2-Z coordinates are poorly predicted.

Furthermore, the accuracy of the learning model is evaluated using the True Positive Rate (TPR or Sensitivity) and the True Negative Rate (TNR or Specificity) measures. The accuracy is defined as the ability of the learning model to predict actual TP-outliers. In this case, measuring the specificity is not relevant as the number of actual TP values within limits is significantly higher than actual outliers. Therefore, the focus is on the ability of the model to correctly predict actual TP outliers. As the model can detect outliers in nearby areas, the definition of a *True Positive* is expanded to include predicted outliers that will actually occur within the next 24 h. A *False Positive* is defined as when no actual TP outlier occurs during the next 24 h, and a *False Negative* is defined when there is an actual TP-outlier that is not predicted within the previous 24 h. Equations (6), (7), and (8) define TPR, False Negative Rate (FNR), and Threat Score (TS), respectively.

6 $$ TPR = {\text{ }}\frac{{{\text{True}}\;\;{\text{Positives}}}}{{{\text{True}}\;\;{\text{Positives + False}}\;\;{\text{Negatives}}}}{\text{ }} $$

7 $$ FNR = \frac{{{\text{False}}\;\;{\text{Negatives}}}}{{{\text{False}}\;\;{\text{Negatives + True}}\;\;{\text{Positives}}}} $$

8 $$ TS = \frac{{{\text{True}}\;\;{\text{Positives}}}}{{{\text{True}}\;\;{\text{Positives + False}}\;\;{\text{Negatives + Fale}}\;\;{\text{Positives}}}} $$

**Table 1:** Accuracy of the learning model for detecting TP outliers within a 24-hour interval

Hole	#. Outliers	*TPR* (%)	*FNR* (%)	*TS*
H1	12	30	70	0.27
H2	0	0	0	0.00
H3	4	50	50	0.50
H4	1	100	0	1.00
H5	1	0	100	0.0

Table 1 reports the number of outliers, *TPR*, *FNR*, and *TS* rates for each hole. The performance of holes H4 and H5, while noteworthy, is not significant since they are only related to a single outlier that the model either predicts well or badly. For hole H1, Table 1 indicates low *TPR* and *TS* rates, which is a result of some predictions and actual TP outliers being separated by more than 24 h. For instance, the predicted TP outlier at observation 200 is separated by more than 24 h from the actual TP outliers at observations 213-215, and similarly with the predicted TP outlier at observation 272 and the actual outliers at subsequent observations. In the case of hole H3, the predicted TP outlier at observation 226 is correctly reported by the learning model; however, the next actual outlier at observation 232 occurs after 24 h, explaining the relatively low TS score obtained for H3. Overall, Table 1 confirms that the learning model cannot accurately predict TP outliers. Nonetheless, the predicted information can still be used by the manufacturer to make early adjustments.

## Conclusion

This paper deals with a prediction problem for quality control. The underlying problem is related to the automotive industry, and the product under study is the bumper beam, subject to stringent quality criteria. To support the quality control process of this product, we proposed machine learning models to predict the location of the reference holes of the next produced beam. The models are based on a time series that consisting of the historical data set of previous measurements that includes the beam characteristics. The learning models developed are a neural network, a long short-term memory network, and a random forest, and all are trained under similar conditions. The experimental study showed that the performance of all models is generally quite similar, with a slight dominance of the long short-term memory network and the random forest models. The results also indicate that the prediction can be good for some hole-coordinate pairs. However, there are considerable discrepancies for some other coordinates, and the predictions deviate significantly from the actual values. Since both models showed similar behavior, it can be concluded that the available information is not sufficient for prediction and that other resources should be included, such as process parameters or data from an upstream activity.

This work shows that applying machine learning models to real-life problems is not as easy as it sounds and is hampered by several factors. Not all data is captured or made available to be used for other purposes. For example, in the context of this work, information about changes in CNC settings is volatile and cannot be retrieved later, limiting its use for learning purposes. This example also shows that the transition to Industry 4.0 is not a straightforward process and could be challenging in several areas.
